# Mobile Phone-Based Intervention in Hypertension Management

**DOI:** 10.1155/2019/9021017

**Published:** 2019-04-07

**Authors:** Nobian Andre, Retno Wibawanti, Bambang Budi Siswanto

**Affiliations:** ^1^Faculty of Medicine, Universitas Indonesia, Indonesia; ^2^Department of Community Medicine, Faculty of Medicine, Universitas Indonesia, Indonesia; ^3^Department of Cardiology and Vascular Medicine, Universitas Indonesia, Indonesia

## Abstract

Hypertension is known as the major risk factor for cardiovascular mortality and morbidity. Antihypertensive agents are directed to prevent many of the harmful effects of elevated blood pressure, yet medication nonadherence hinders the effectiveness of these therapies. Nowadays the use of mobile phone has vastly spread among communities. The rapid adoption of smartphone technology creates a promising and interesting platform to overcome medication nonadherence. This review aimed to critically appraise whether mobile phone-based interventions are effective in increasing adherence in hypertensive patients. Literature searching was done in 3 databases: PubMed, Cochrane, and ProQuest. Findings were narrowed down using selection criteria. Relevant studies were to be critically appraised based on the guideline from Centre for Evidence-Based Medicine, Oxford University. We found that the reduction of blood pressure in participants who were given reminder through mobile phones was greater in comparison to control: systolic (94.4% vs 41.2%, p 0.003), diastolic (94.4% vs 76.5%, p0.04). Patients who were nonadherent at baseline benefit more from mobile phone-based intervention in comparison to adherent patients at baseline: RR 2.3 (95% CI: 1.4-4.4, p<0.001) vs RR 1.3 (95% CI: 1.0-1.6, p<0.05). In conclusion, mobile phone-based interventions were effective in increasing medication adherence in hypertensive patients. Clinical practice guidelines should consider this nonpharmacological method for a better blood pressure regulation.

## 1. Introduction

Hypertension is defined as a systolic blood pressure greater than 140 mmHg and a diastolic blood pressure greater than 90 mmHg from an average of at least 2 measurements [1]. The prevalence of hypertension, based on the measurement of adult population (≥18 years old), reached 34.1% in Indonesia in 2018 [2]. Furthermore, 45.6% of these patients do not routinely take their medication. Most of them (59.8%) do not feel any symptoms while some others often forget [2]. Globally, there are 9.4 million deaths caused by hypertension through its effects on cardiovascular health every year [3]. Hypertension is known as the main risk factor for cardiovascular morbidity and mortality. In addition, this condition is a major contributor to the health and economic burden imposed by stroke, heart disease, and renal insufficiency [1]. Complications can occur because the relationship between blood pressure and the risk of cardiovascular events is continuous, consistent, and independent of other risk factors [1]. Antihypertensive agents are directed to prevent many of the harmful effects of elevated blood pressure, yet medication nonadherence hinders the effectiveness of these therapies [3]. As in patients with other chronic diseases, medical adherence is a major problem faced by hypertensive patients. Poor blood pressure control has a negative impact on the course of hypertension by causing various complications. Nowadays the use of mobile phones has vastly spread among communities. It is said that the cellphone is the fastest adopted technology in low- and high-income countries [4]. Therefore, a new model of health approach through cellphones is increasingly being used [4]. The rapid adoption of smartphone technology creates a promising and interesting platform to overcome medication nonadherence by providing drug intake reminders, offering healthy lifestyle education, or keeping records of biometric measurements [3]. Several past studies have examined the effect of mobile phone-based interventions on medication adherence in hypertensive patients. This review aims to critically appraise whether mobile phone-based interventions are effective in increasing adherence in hypertensive patients.

### 1.1. Case Report

A 62-year-old woman came to a clinic with a worsening headache for the past 3 days. The pain was felt continuously, such as being pressed on the back of the head, was not pulsatile, and was of moderate intensity. Complaints of nausea, vomiting, decreased consciousness, visual disturbances, slurred speech, or body weaknesses were denied. A history of head trauma was also denied. The patient said that such complaint was often experienced during high blood pressure. The patient was diagnosed with hypertension 10 years ago. She had been given antihypertensive drugs but did not consume them routinely. Her highest blood pressure was 180 mmHg with an average of 150–160 mmHg. She usually visits the clinic and takes her medication only when complaints are present. She does not regularly take her medication because she often forgets and feels no symptoms. In physical examination, her blood pressure was 150/100 mmHg; other vital signs were within normal limits. Visual Analogue Scale (VAS) was 6–7 at the occiput region, with no tenderness nor inflammation. Other physical examinations were within normal limits.

### 1.2. Clinical Question Formulation

Based on the presented case illustration above, we formulate a clinical question, “How does mobile phone-based intervention affect the medication adherence of hypertensive patients?”

Hence, the PICO framework derived from the question above is as follows:

- Patient(P): hypertensive patient
- Intervention (I): mobile phone-based intervention
- Comparison (C): standard care
- Outcome (O): adherence

## 2. Method

### 2.1. Search Strategy and Selection Criteria

**Figure 1:**
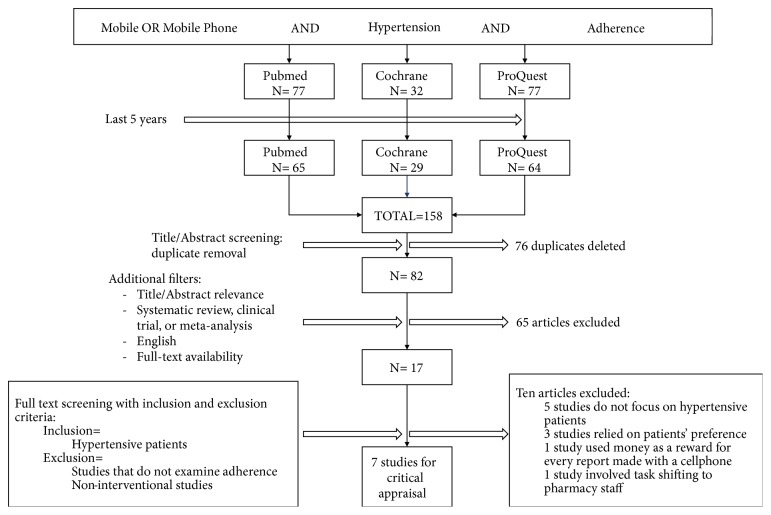
Selection flow chart of the literature review search.

Data for this review were identified through searches. Literature searching was performed in 3 databases: PubMed, Cochrane, and ProQuest on December 15th 2018. Only articles published in the last 5 years were included. The terms found in PICO were formulated using Boolean technique to be used as keywords in each database (Table 1). Subsequently, search yield was narrowed down through a selection process depicted by Figure 1. Relevant articles were appraised based on the critical appraisal guideline by Centre for Evidence-Based Medicine (CEBM), Oxford University, obtained from http://www.cebm.net/critical-appraisal/.

## 3. Result

### 3.1. Critical Appraisal

In this review, we examined the efficacy of mobile phone-based intervention in increasing medication adherence in hypertensive patients. We found 158 records using the search strategy noted in Table 1 and finally identified 7 articles that matched our criteria: 5 clinical trials and 2 systematic reviews. Critical appraisal was carried out on all selected articles (Tables 2 and 3).

In terms of validity, the study by Kim et al. had a low validity value due to the absence of blinding technique; hence treatment allocation was disclosed although participants had already been randomized. In addition, out of 160 participants who were randomized, only 95 participants left at the end of trial (loss to follow-up >20%). Therefore, this is not in accordance with the intention-to-treat principle. The remaining 4 clinical trials were considered valid (study by Davidson et al. fulfilled at least 4 out of 6 criteria). Two systematic reviews were also considered valid.

In terms of importance, 3 studies had low scores. Those include studies by Davidson et al., Kim et al., and Bobrow et al. In the study by Davidson et al., the data of control group was less presented than the intervention group. Consequently, its clinical importance and the statistical significance were not clear. In the study by Kim and colleagues, the intervention did not bring about a large nor significant effect in treatment group when compared to the control group. However, note that their study participants were taken from hospital employees and their relatives who had better health knowledge and considerably adherent background. Thus, the study results may not reflect that of general population and its clinical importance remains uncertain. The results of the study from Bobrow et al. did not display a robust enough evidence due to the wide data distribution (poor precision) so that poor adherence was also found in the intervention group. In other words, their intervention did not succesfully increase patients' adherence. The remaining 2 studies had clinically important results.

In terms of applicability, the most practical study—relating to the case illustration—would be the study by Varletta et al. on the basis of the similarity of basic characteristics of the study population (most were women, aged 60, it included patients with low educational level, and baseline systolic blood pressure is ± 140 mmHg) and the type of intervention that was in the form of text messages (simple). In contrast, the studies most difficult to apply to our patient would be those of Davidson and Kim due to the fact that their intervention involved monitor devices connected to mobile phones (less practical) and the high educational background of their participants.

### 3.2. Types of Mobile Phone-Based Interventions

There were various forms of interventions: from the conventional text messages (SMS) to smartphone applications, and a more sophisticated method that involved an external monitoring device. Bobrow et al. utilized text messages to provide medication motivations along with education about hypertension and its treatment [8]. Varletta et al. added some additional features in their text message intervention such as education about the importance of medication intake and adherence, educational information about healthy diet, and also antihypertensive medication schedule [6]. Another team designed a smartphone application that provided educational information about hypertension, a drug intake reminder, and a routine clinic visit reminder. This application also stored blood pressure measurements, blood pressure target, and physician's note on the patient's antihypertensive [9]. Davidson et al. had a slightly different approach whereby they utilized a smartphone application that sent reminders (drug intake and blood pressure monitoring) every 3 days and it was linked to an external monitoring device [5]. Kim et al. used similar means [7].

### 3.3. Outcome Measures

The primary endpoint is medication adherence after mobile phone-based intervention. Another outcome of interest is blood pressure changes as a result of medication adherence. Based on literature, medication adherence is defined as the degree to which a patient follows the prescribed dosage, frequency, and timing of drug intake [5]. To measure the level of adherence, studies used a variety of parameters such as pharmacy refill rate [8], the timing of the opening of a drug container lid (with embeded microchip) [9, 10], independent blood pressure measurements [5], and the result of patients' blood pressure reduction [5, 10, 11] or through a Morisky adherence questionnaire [6, 7]. The follow-up periods varied from 3 months [10] and 6 months [5–7] up to a year [8–10]. There is a tendency of the study results to exhibit medication adherence improvement after mobile phone-based intervention. Some even showed a significant increase in adherence.

## 4. Discussion

Poor blood pressure control has a negative impact on the course of hypertension by causing various complications. Antihypertensive agents are directed to prevent many of the harmful effects of elevated blood pressure, yet medication nonadherence hinders the effectiveness of these therapies [3]. This review aimed to critically appraise whether mobile phone-based interventions are effective in increasing adherence in hypertensive patients. We identified 7 articles that matched our selection criteria to be critically appraised. From critical appraisal we found that the study by Kim et al. [7] was not valid since it only fulfilled 50% of the validity points of the guide. Therefore, their study intervention and results will not be taken into consideration. Another study data by Davidson et al. [5] was incomplete, making it difficult to determine its clinical importance in the aspect of medication adherence (but their data was sufficient for blood pressure reduction). Next, the study by Bobrow et al. [8] did not show evidence of a strong intervention since their results were not clinically important.

### 4.1. Mobile Phone-Based Intervention in Hypertension Management

Overall, studies found a tendency of increased medication adherence levels in hypertensive patients as a result of mobile phone-based interventions. Although the types of interventions and adherence parameters vary across studies, the results are in line with one another. This enhances the value of an intervention because the results are consistent when measured using various criteria.

Mobile phone-based intervention can be divided grossly into 2 types: text messages and smartphone applications. Both have the role of providing a drug intake reminder, an independent blood pressure monitoring reminder, or a routine clinic visit reminder. This is crucial given that it is not uncommon to encounter hypertensive patients who take their drugs and pay a clinic visit only when complaints are present, making preventive roles of antihypertensives inefficient. After studies are conducted, it was found that patients who were given reminders had better blood pressure control [5, 10].

**Figure 2:**
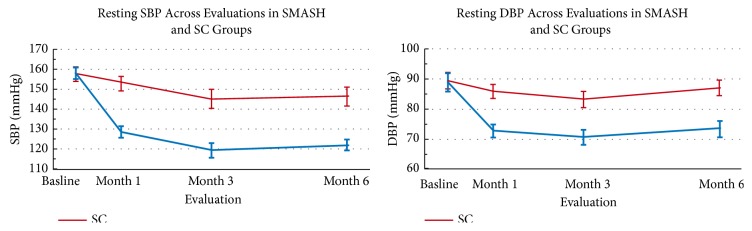
Blood pressure control was better in the intervention group (blue) in comparison to control (red). SBP: systolic blood pressure; DBP: diastolic blood pressure; SC: standard control; SMASH: smartphone medication. Source: [5].

**Figure 3:**
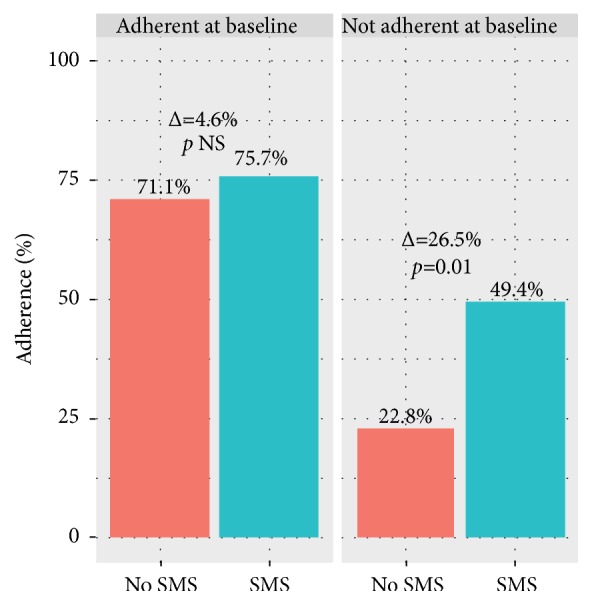
The increase in proportion of adherent participants after text message reminders is greater in the nonadherent at baseline group. Source: [6].

There are differences in the frequency of sending drug reminders between studies: at every dosing time [10], every day [5], or every 12 ± 2 days [6]. However, the study that gave reminders every 12 ± 2 days did not take measurements of blood pressure, and their medication adherence parameter was different from the other 2 studies. Therefore, a fair comparison of the three cannot be done. Decreased blood pressures were clearly shown after participants received reminders every day (Figure 2) and at every dosing time [5, 10]. Reminders at every dosing time also proved to be helpful in helping patients as indicated by the increase of drug intake action (from microchip MEMS) [10]. Furthermore, patients who were nonadherent at baseline benefit more from mobile phone-based intervention in comparison to adherent patients at baseline (Figure 3) [6]. An increase in medication adherence would be very helpful for physicians in making medical decisions as it enables blood pressure measurement to reflect true condition of the patient so that additional doses or drugs can be given if a good blood pressure control has not been achieved.

Aside from medication adherence, blood pressure control is also influenced by other factors including drug dose, antihypertensives agent, and lifestyle. Therefore, in addition to a reminder feature, mobile phone-based intervention can also be complemented by other features such as motivational messages, educational information regarding healthy lifestyle for hypertensive patients, the importance of consuming drugs regularly, or educational information regarding hypertension in general [6, 8, 9]. Text messages can be put on based on the values and beliefs of a patient [5]. As an example, a grandmother would receive a text message saying: “Taking medicine is good, taking it at the right time is better! Your grandkids need you in their future” [5].

In the form of an application, several additional features—like keeping a record of blood pressure measurements, displaying a tailored blood pressure target, and physician's note on the patient's antihypertensive [9]—can be added on top of its reminder feature. Note that the drug intake reminder feature has a main role. This is shown by the results of 2 studies in which the intervention did not involve drug intake reminder so that the reduction of blood pressure was not clinically important and the adherence levels intersect between control and intervention groups [7, 8].

### 4.2. Implications for Clinical Practice

Translating this review into clinical practice, we recommend a study to be conducted on the subject of mobile phone-based intervention efficacy in hypertensive patients in Indonesia prior to applying such therapy to patients given the absence of similar intervention technique in Indonesia. Furthermore, future research should also focus on evaluating the appropriate reminder frequency and the type of intervention that is best applied. It is worth noting that the studies by Davidson et al. [5] and Kim et al. [7] had less applicable interventions (esp. in the setting of a developing country) as they require an additional monitoring device aside from the mobile phone. Such method should be avoided. Furthermore, mobile phone-based intervention is recommended to also provide educational information regarding lifestyle modification (i.e., diet and exercise suitable for hypertensive patients) in addition to adherence interventions.

## 5. Conclusion

Mobile phone-based interventions were effective in increasing medication and blood pressure monitoring adherence in hypertensive patients. Given the high burden of uncontrolled hypertension worldwide, this nonpharmacological strategy could be considered as an adjunct to antihypertensive medication. Nonetheless, such intervention should be developed further before being applied in daily practice.

## Figures and Tables

**Table 1:** Keywords and number of findings according to the databases (conducted on December 15th 2018).

Database	Search terms	Hits	Selected article(s)
PubMed	(mobile phone [Title/Abstract] OR mobile [Title/Abstract]) AND hypertension [Title/Abstract/MeSH Terms] AND adherence [Title/Abstract/MeSH Terms]	77	3

Cochrane Library	mobile phone [Title/Abstract/Keyword] AND hypertension [Title/Abstract/Keyword] AND adherence [Title/Abstract/Keyword]	32	2

ProQuest	noft(mobile phone) AND noft(hypertension) AND noft(adherence)	77	2

**Table 2:** Critical appraisal of therapeutic studies.

Author	Year of publication	Study design	Number of participants	Level of evidence	Validity						Importance			Applicability		
					Randomization	Allocation concealment	Intention-to-treat	Blinding	Comparable treatment	Similarity treatment & control	Clinical importance	Statistical significance*∗*	Precision of Treatment effect	Domain	Feasibility of treatment	Benefit overweighs harm
Davidson et al. [5]	2015	RCT	38	2	+	?	+	?	+	+	?	?	+	-	-	+
Kim et al. [7]	2016	RCT	95	2	+	-	-	-	+	+	-	-	+	-	-	+
Bobrow et al. [8]	2016	RCT	1157	2	+	+	+	+	+	+	-	+	-	-	+	+
Varleta et al. [6]	2017	RCT	291	2	+	+	+	+	+	+	+	+	+	+	+	+
Contreras et al. [9]	2018	RCT	148	2	+	+	+	+	+	+	+	+	+	-	+	+

+ means present; - means absent; ? means unclear/not mentioned; *∗* means statistical significance at p<0.05.

**Table 3:** Critical appraisal of systematic reviews.

Author	Year of publication	Study design	Level of evidence	Validity				
				PICO suitability	Appropriate searching	Relevant study included	Quality assessment of trials	Heterogeneity
Gandapur et al. [10]	2016	Systematic review	1	-	+	+	+	-
Xiong et al. [11]	2018	Systematic review	1	+	+	+	+	-

+ means present; - means absent; ? means unclear/not mentioned.
